# Virtual reality-based medical education versus lecture-based method in teaching start triage lessons in emergency medical students: Virtual reality in medical education

**DOI:** 10.30476/JAMP.2021.89269.1370

**Published:** 2022-01

**Authors:** SAMAN BEHMADI, FARIBA ASADI, MARYAM OKHOVATI, ROGHAYE ERSHAD SARABI

**Affiliations:** 1 Management and Leadership in Medical Education Research Centre, Kerman University of Medical Sciences, Kerman, Iran; 2 Ferdows Paramedical School, Birjand University of Medical Sciences, Birjand, Iran; 3 Medical Informatics Research Center, Institute for Futures Studies in Health, Kerman University of Medical Sciences, Kerman, Iran

**Keywords:** Virtual reality, Lecture, Simulation, Education, Triage, Emergency, Medical students

## Abstract

**Introduction:**

Epidemics such as the recent one, COVID-19, have caused a change in education and its methods. Virtual simulation is one of the types of technology used in medical education and related sciences. Virtual simulation provides practical and clinical medical education in a safe, cost-effective, reproducible, and flexible learning environment where students can practice over and over in a standard environment. This study was conducted to compare the effect of virtual-based medical education versus lecture-based method in teaching start triage lessons in emergency medical students in educational and medical centers affiliated to Birjand University of Medical Sciences Ferdows, Paramedical School, in 2020.

**Methods:**

This is a quasi-experimental study. َAll 44 students of emergency medicine registered for triage course participated in the study. They were divided into two homogenous groups which were matched based on their grade point average. The simple triage and rapid transport (START) triage course was taught in the traditional way- lecture based- from the beginning of the semester to the middle of the semester; then, the other group (simulation-based) was trained in the second half of the same semester using virtual simulation.

**Results:**

The students’ rate of learning was measured by their scores at the end of the course exam in both groups. The mean scores of virtual simulation-based education were slightly higher than those of the lecture-based education, but it was not statistically significant (P>0.05). The students were more satisfied with virtual simulation-based education than the lecture-based and the difference between the mean scores of satisfaction was statistically significant (P>0.05).

**Conclusion:**

The results of this study suggest that VR can effectively improve knowledge in undergraduate emergency student’s education, but it was not more effective than traditional educational methods. More experimental studies with a larger sample size are needed to confirm that virtual simulation-based education can more effectively improve knowledge in teaching practical lessons such as triage.

## Introduction

One of the most prevalent teaching methods in medical science curriculum is lecture-based teaching. Simplicity, suitability for crowded classrooms, the huge size of educational materials, and time limit are some features of lecture-based teaching and this method is teacher-centered ( 1 ). Although this method is widely used, students are passive in such classes ( 2 ). In this way, medical education which focuses on small groups and case-based scenarios is rapidly evolving ( 3 ). New methods have recently been offered for better training of medical students, and universities should apply these methods to train students capable of preventing diseases, treating patients, and promoting community health ( 4 ). Recent advances such as virtual patients and simulation programs have facilitated an active teaching approach ( 5 , 6 ). Nowadays, educators using simulation training can deliver medical education with new approaches. Simulation-based medical education has developed during the past 40 years ( 7 ). It is a method by which an artificial experience is created that engages the learner in real-life situations without dangerous or harmful situations ( 8 ). This training method has been widely used in many areas of medical education, especially those requiring practical work. *Some evidence has shown that* virtual reality simulation in the acquisition of clinical psychomotor skills leads to similar or superior educational outcomes in comparison to traditional simulated practice ( 9 ). A systematic review by Harder has shown that the use of simulation, compared to other educational methods, improves the skills of health care students ( 10 ). Another study conducted by Luigi Ingrassia in Italy (2015) on virtual simulation training and real model training on emergency room attendance patients indicated that simulation training increases the students' accuracy in initial START triage. It was also shown that students' abilities and speed of action before and after the application of virtual simulated models have changed significantly ( 11 ). Also, in 2018, Harrington et al. taught decision-making skills to severely injured patients in a trauma department. Participants reported that this was an enjoyable as well as cost-effective learning tool ( 12 ). Some other systematic reviews have evaluated the effect of simulation-based medical education in some specialties and sub-specialties such as radiation oncology ( 13 ), echocardiography ( 14 ), emergency medicine ( 15 ), and trauma education ( 16 ).

Simulation training has been proven to develop the students’ clinical decision-making abilities and improves confidence and satisfaction ( 16 ). In fact, medical and paramedical students need to update their knowledge and skills. In this way, educational methods should be used which strengthen their self-learning, reasoning, and judging ( 17 ). On the other hand, the outbreak of COVID-19 virus in 2020 has influenced medical education. We have to adapt ourselves to this new situation to prepare future clinicians for their professional services ( 18 ). In this regard, virtual learning is widely used in medical education around the world, so it seems there is a shift from simulation-based to virtual simulation-based learning ( 19 ). Despite the evidence, the application of any new educational method in different educational environments is associated with challenges ( 20 ). In this way, simulation-based medical education should be thoughtfully and cautiously introduced and evaluated ( 21 ), especially considering the fact that it engages people’s life. 

Emergency medicine is a relatively young field, but it has been quick to use simulation technology ( 15 ). Previous research has studied simulation in emergency education ( 22 , 23 ). Studies have demonstrated the effectiveness of simulation in teaching basic sciences, clinical knowledge, procedural skills, teamwork, and communication skills. As simulation becomes increasingly prevalent in medical school curricula, more studies are needed to assess whether simulation training improves the patient-related outcomes ( 15 ); its effect should be studied and considered, so that its challenges and advantages are identified and . Although lecture-based teaching is widely used, it is not the same for all learning styles ( 24 ); in all situations, the lecture-based teaching has been reported to significantly improve the performance in comparison with the teach-back and the concept map groups, while these two significantly improve knowledge ( 25 , 26 ). Therefore, different teaching methods should be studied.

This study was carried out to evaluate the effectiveness of learning through virtual simulation in comparison with traditional method in learning of START triage knowledge and skill level in a group of undergraduate students majoring in the field of emergency medicine at Birjand University of Medical Sciences, Ferdows Paramedical School, from September 2019 to January 2020.

## Methods

The present quasi-experimental study was performed on two groups to compare the effect of virtual simulation-based and lecture-based training on students' learning in triage course. The census sampling method was used in this study. The study population consisted of all 44 emergency undergraduate students who were enrolled in the triage course in 2019-2020 at Ferdows Paramedical School. The students were divided into two homogeneous groups based on their age and grade point average and then randomly assigned to traditional lecture or virtual reality-based learning groups (22 in the intervention group and 22 in the control group). The first group received lecture-based training and 22 students in the second group who received virtual simulation-based training; when allocating the students in each group, their gender and grade point average (GPA) were considered to control their effect, so the groups were homogeneous. Then, the semester was divided into two parts. In the first part of the semester, according to the pre-determined schedule, the first group attended the classes and the lectures were presented, from the beginning of the semester to the middle of it. Then, the second group was taught using virtual simulation-based from the middle to the end of the semester. The content was the same for both groups and the same teacher prepared it. Virtual reality simulation videos were shown to the students in the virtual simulation group in the “crisis management room” of Ferdows Paramedical School, which was equipped with LCD TV and virtual reality glasses. The most appropriate films were used according to the lecture title and according to the triage guideline and triage protocol through START (Simple Treatment and Rapid Transport) method, which is approved by the Ministry of Health (1374) and notified by the emergency department of 115 countries. The content used was simulated "START method triads and its features" according to the course topics presented in the lecture group. To evaluate and compare the efficiency and level of education of these two methods, the students’ scores in the exam of this course were used; also to assess the students’ views on each of these methods, a self-assessment questionnaire with 7 questions was completed at the end of the course on a 4-point Likert scale (4 = excellent, 3 = good, 2 = Fair, 1 = poor).  This questionnaire was completed by the intervention group at the end of the exam. The validity of the questionnaire was determined through several discussions and revisions by the three members of the faculty in departments of medical education. The reliability of the questionnaire was evaluated using internal consistency; the Cronbach coefficient was 0.83. After examining the data for accuracy and the absence of outlier, data Kolmogorov-Smirnov test was used to examine the normal distribution of data. To compare the two teaching methods of lecture and simulation, analysis of covariance was used by considering the GPA confounder variable. The presumption of the equality of variance between the groups was confirmed using Leven’s test.

### Ethical Consideration

This study was approved by ethics committee of Kerman University of Medical Sciences, with the code of IR.KMU.REC.1399.172.

## Results

In this study, 44 participants with a mean age of 21.13±1.11 were enrolled. All of them were male; among them, 72.7% were single, and the others were married. The results of normality showed that the score variable was normal, but the evaluation variable was not normal. Table 1 shows the mean score and variance of the participants in the two methods; according to this Table, the mean score of teaching based on virtual simulation was higher than that of the lecture group.

**Table 1:** Description of the mean and variance of the score of the participants in the two groups

Teaching method	Frequency	Mean±SD
Lecture-based	22	16.67±1.82
Simulation-based	22	17.32±1.83

According to the results of the analysis of covariance ( Table 2), the difference in the scores between the two groups was not significant. In other words, the training method did not have much effect on the score of the participants. According to the beta values, the score of those who had experienced the simulation training method by controlling the grade point average effect was 0.47, which was higher than those who had used the lecture method. There was no significant difference between the two groups based on their GPA (p = 0.99).

**Table 2:** Results of analysis of covariance for comparison of the scores between the two groups of lectures and simulations

Variable	Beta	SE	t	p
Training method	-0.47	5.68	0.007	0.93
GPA	0.53	0.24	9.88	0.003
Teaching method ^*^ GPA	-0.004	0.34	0.000	0.99

The results showed that the participants were more satisfied with the simulation training method, and the difference in the satisfaction score was significant. The mean of satisfaction in the lecture method was a little higher than moderate, while it was good for simulation-based group ( Table 3).

**Table 3:** Comparison of the participants’ satisfaction mean scores between the lecturing and virtual simulation-based education

Group	Mean±SD	Mann-whitney U	p
Lecture	3.36±0.31	8	0.001
Simulation	4.10±0.20

## Discussion

The present study was an attempt to investigate the effect of virtual simulation-based training on learning triage lessons among emergency medical students in comparison with lecture teaching. The findings of this study, considering the comparison of the mean scores of the two educational groups, showed that virtual simulation-based education helped the instructors meet the expectations related to learning to some extent more than the lecture method. In this regard, Luigi Ingarsia’s study compared virtual reality simulation with live simulation to test the ability of 56 medical students in two groups; he performed a triage of casualties using the Simple Triage and Rapid algorithm after training, showing similar results. Although there was no significant difference in the results, it was concluded that virtual reality simulation was a valuable tool, equivalent to live simulation, to educate and evaluate the ability of medical students to perform triage of casualties and detect progress in such skills ( 11 ). 

Also, the results of the study conducted by McGrath et al. (2017) which examined the consensus of experts on the use of virtual simulation in teaching emergency medicine were in line with the findings of this study. This study confirmed the need to create more focused environments and the use of advanced technologies for medical evaluation and education ( 22 ). 

The study carried out by Tobloo et al. evaluated virtual simulations in theoretical learning and laboratory performance of dental students. Contrary to the results of our study, it showed a significant effect on the students' learning, and those who used virtual learning achieved better results than control students ( 27 ). 

Amir Alavi et al. compared the effect of using a bronchoscopic web-based simulator and traditional education methods on the knowledge of tracheobronchial anatomy of anesthesia assistants in Guilan University of Medical Sciences. The results of this study showed that bronchoscopy training through simulator increased the learning rate of residents more than the traditional method ( 28 ). 

Emami Sigaroudi et al. have obtained completely different results and reported that there was a significant difference between the two training methods. The scores in the traditional approach were higher than the electronic method; they reported that traditional education method was superior to electronic education ( 29 ). 

According to Hashemi et al., comparing the effect of the two methods of teaching "lecture" and "simulation using the patient" in improving the knowledge and practice of health care providers, no significant difference was observed between the two groups ( 30 ). 

Finally, a systematic review study put forward the same results as the findings of this study. In the 2018 study, Khan examined whether virtual reality simulation training could be used as a complement to and/or alternative to patient-based introductory gastrointestinal endoscopy. They did not find virtual reality to be superior to traditional patient-based education or any other method of endoscopic simulation training. They reported that the existing virtual reality simulation programs could be improved using educational theories such as gradual learning strategy, where trainees increasingly complete difficult tasks. The results of this review showed that endoscopic virtual reality training could be used to supplement conventional endoscopic basic training for trainees with limited or no endoscopic experience ( 31 ).

### Limitation

This study had some limitations; the short time of the courses was the most important limitation. The participants were from the same class, the exchange of information between them could be an important limitation that was considered. This study was conducted only on emergency medical students of Ferdows city in the academic year of 2019-2020 and these should be considered when generalizing its results.

## Conclusion

Virtual simulation has several advantages. According to the results of this study, a virtual simulation-based training method, like traditional training, can achieve the goals of the training program. This method leads to an increase in students' knowledge and performance (skills) in the field of triage lessons. Simulation-based education is a useful educational method that can surpass traditional methods in improving academic performance and increase the effectiveness. As it mimics the real life, in the pandemic condition in which virtual teaching is widely used, it can highly be recommended. According to this study, the students were more satisfied with virtual simulation-based education than lecture-based, so it is suitable for practical lessons such as triage. 

## Acknowledgement

The authors would like to acknowledge all the emergency students who participated in this research.

**Conflict of Interest:** None Declared. 
